# Economic deprivation and intimate partner violence in Germany

**DOI:** 10.1371/journal.pone.0329930

**Published:** 2025-08-18

**Authors:** Lara Minkus, Ruth Abramowski

**Affiliations:** 1 Department of Social Stratification, Empirical Methods and Statistics, Europa-Universität Flensburg, Flensburg, Germany; 2 SOCIUM Research Center on Inequality and Social Policy, University of Bremen, Bremen, Germany; Anglia Ruskin University, UNITED KINGDOM OF GREAT BRITAIN AND NORTHERN IRELAND

## Abstract

This article examines the association between economic deprivation and the likelihood of experiencing physical intimate partner violence (IPV) in Germany. Although international studies have shown a link between economic deprivation and IPV, analyses utilizing probability-based surveys to examine this association in Germany are scarce. Within the framework of an economic power resource approach and using the German Family Panel *pairfam*, a large probability-based panel, this study aims to fill this gap. Results of linear probability models show that being unemployed and being unsatisfied with the household finances, as indicators of economic deprivation, significantly increase the risk of physical IPV. A range of sensitivity checks corroborate these findings.

## Introduction

Intimate partner violence (IPV) and especially violence against women is a global phenomenon. Findings from the World Health Organization [1] estimate that about 30 percent of women worldwide are survivors of physical and/or sexual partner violence or non-partner sexual violence in their lifetime. Intimate partner violence is the most common type of violence. The results of a systematic review and meta-analysis of 366 studies from over 160 countries show that, globally, 27% of women aged 15–49 who have ever been in a relationship have experienced physical or sexual violence – or both – from a current or former partner at some point during their lifetime [2]. In Germany, recent figures indicate that every third woman has experienced physical and/or sexual violence at least once in her life and about every fourth woman has experienced physical or sexual violence at least once by her current or former partner [3].

Inequalities in women’s economic power, such as the unequal distribution of care work and gender pay and wealth inequalities, are often seen as risk factors for violence against women. However, this link remains theoretically and empirically under-researched and the empirical evidence on this topic is ambiguous [4]. On the one hand, a large body of literature found a positive relationship between economic powerlessness and IPV [e.g., 5–8,9]. On the other hand, some studies find no or no strong relationship, or an increased risk for women with high educational and social resources [e.g., 10–12]. In Germany, the lack of probability-based surveys that include items on IPV undoubtedly contributes to this ambiguity found in the empirical evidence on the topic. This article sets out to fill this gap. We use data from the German Family Panel (*pairfam*), a nationwide probability-based panel that also includes questions on IPV.

This article builds on the assumption that economically deprived individuals have limited bargaining power. Specifically, we examine how economic deprivation is linked to physical IPV in Germany. Women who are economically dependent on their partner often have limited options to leave violent relationships – for example, they may be unable to afford housing for themselves or financially support their children independently. Emerson’s [13,14] power-dependency model highlights the relationship between interdependence and power. The model suggests that power imbalances in relationships arise from two conditions: first, the availability of alternative exchange relationships, which provide the dependent individual with power by reducing reliance on the current relationship [see also 15]; second, the possession of resources, which is key to acquiring power [see also 16]. The more resources a person possesses, the more alternatives are available to that person [17]. In this context, we conceptualize economic deprivation – operationalized through unemployment and dissatisfaction with household income – as indicators of economic powerlessness. It is worth noting that physical violence is not the only type of violence that can occur in a relationship. Sexual, economic and emotional abuse are also prevalent. All these forms are under-researched, especially from a quantitative social science perspective. Nevertheless, this article focuses solely on physical violence within partnerships. This study is thus among the few to examine the association between economic deprivation and the risk of physical IPV in Germany, using data from a probability-based panel (for a notable and recently published exception see [9]).

The article is structured as follows: First, the current state of the art is outlined, accentuating the research gap in violence, work, and the socio-economic situation of couples in Germany. Second, the theoretical discussion on IPV is highlighted from an economic power resource approach. Third, the article provides an empirical investigation of the impact of economic deprivation on IPV against women in Germany. Last, the article concludes with a discussion of the results.

## State of the art

Intimate partner violence remains a widespread issue in Germany. During the COVID-19 pandemic for example, IPV cases in Germany have risen significantly, with women accounting for 80.1% of survivors in 2021 [18]. Regional studies further underscore the severity of the issue; for example, in Saxony, 30% of participants reported experiencing sexual coercion, predominantly by male partners in domestic settings [19]. Research also suggests that major life transitions – such as cohabitation, marriage, parenthood, divorce, or career changes – increase women’s vulnerability to violence [20,21].

A critical factor of IPV is socio-economic deprivation. Steinert and Ebert [22] found that households with young children, especially those affected by unemployment or short-time work during the COVID-19 pandemic, reported higher rates of IPV. Similarly, Jud et al. [23] identified associations between IPV and characteristics such as being female, older age, unemployment, poverty, and prior IPV perpetration. Despite these findings, research on the intersection of work, socio-economic conditions, and IPV in Germany is limited, with few studies adopting an inequality perspective (but see [9] and for descriptive evidence see [24]). Family sociology, too, has largely overlooked this issue [25], despite the family being a high-risk setting for violence [26].

IPV has generally been studied more extensively in public health and criminology than within sociological research on inequality. A notable and very recent exception is Molina et al. [9], who, using *pairfam* data, found that among recently separated women, having minor children increases the risk of IPV, while higher education and employment in the year preceding separation served as protective factors (see also [23]). Furthermore, focusing on IPV perpetrators, Schumann et al. [27] found no correlation between economic hardship (poverty and unemployment) and IPV perpetration after controlling for personality traits and childhood experiences in Germany.

Although research on IPV, work, and economic deprivation in Germany is limited – with few exceptions noted above – international studies (only a selection of which is discussed below) offer some insight, albeit with ambiguous results. From a macro-level perspective, Aizer [28] demonstrates that a decreasing gender wage gap in the U.S. contributed to reduced domestic violence against women. Research from Brazil shows that mass layoffs lead to an increase in domestic violence [29]. At the micro-level, which is the focus of our research, studies from the U.S. [6] and the EU [8] indicate that women with lower education and economic resources face higher risks of IPV. Furthermore, some studies suggest that financial problems and poverty are linked to higher levels of violence in relationships (e.g., [7,30] in the U.S. [31]; in Australia). Conversely, research from Chicago/U.S. [10], the UK [12], and Sweden [10, which is, however, only a descriptive study] suggests that women with higher economic resources may also face elevated risks. These discrepancies likely result from differences in societal contexts, data sources, and research methodologies.

Systematic reviews shed light on the complexities of intimate partner violence. Based on a meta-analysis, Eggers del Campo and Steinert [4] found that women’s economic empowerment – specifically, having more financial resources – leads to a reduction in IPV in most countries. In contrast, McCarthy et al. [32] conducted a systematic review focusing on male IPV perpetration and gender inequitable norms and found that, unlike the findings of Eggers del Campo and Steinert [4], measures of relationship power showed less consistent associations with IPV. Instead, they identified a stronger association between IPV perpetration and attitudes such as the acceptance of violence against women or beliefs about men’s sexual entitlement. However, McCarthy et al. [32] used distinct measures of power and control in relationships – such as behavioral practices, behavior control, decision-making dominance, and dissatisfaction with relationship power – which are not fully comparable to the concept of economic empowerment examined by Eggers del Campo and Steinert [4].

In summary, while international research and systematic reviews offer valuable insights into the relationship between power, economic resources, deprivation, and IPV in different countries (even though with ambiguous results), the German context remains underexplored. Specifically, the interplay of IPV, work, and socio-economic inequality has received little attention. This gap underscores the need for further studies using probability-based large-scale survey data that focus specifically on Germany.

## Theoretical background and hypotheses

### A power resource approach

In the last two decades, family sociological power theories have lost prominence, overshadowed by a growing research focus on gender equality. Many phenomena, such as the Gender Pay Gap, are often framed as issues of gender (in)equality, without addressing the underlying concept of power. This study aims to demonstrate that economic power approaches are crucial for understanding intimate partner violence against women. Since women are disproportionately affected by IPV (in Germany, eight out of ten survivors are women) [18], our study focuses on female survivors. Within the framework of an economic power resource approach, the guiding question is whether economic power deprivation is associated with an increased risk of intimate partner violence against women.

From the 1960s onwards, family sociological power theories emerged and grew in prominence [e.g., 33–37]. While the link between IPV and economic power is not new [38], this perspective has more often been used to explain other aspects of relationship dynamics (e.g., division of household tasks [39–41]), rather than IPV in the German context. Based on Blood and Wolfe’s [33] resource theory, “power may be defined as the potential ability of one partner to influence the other’s behavior. Power is manifested in the ability to make decisions affecting the life of the family” [33]. Economic resources play a crucial role in the power dynamics of relationships, particularly in IPV contexts. Women who are economically deprived often lack power, making them more vulnerable to IPV. Power resource theories suggest that a woman’s ability to leave an abusive relationship is constrained if the household (in particular she) lacks economic resources, weakening her position in negotiations and thus in violent interactions [42,43]. Various economic power approaches, including bargaining theories, argue that increasing a woman’s economic opportunities outside the relationship can enhance her bargaining power and reduce IPV risk [5,44,45]. These approaches are based on Ott’s [46] household bargaining idea, which asserts that the partner with greater resources holds more power. As Farmer and Tiefenthaler [45] argue, women’s earnings and employment status would increase their bargaining power and reduce IPV. Consequently, women with a low income, who are less educated and unemployed, are at a higher risk of experiencing intimate partner violence.

From the survivor’s perspective, indeed, and as already described in the state of the art section, many studies found empirical evidence for a higher risk of experiencing IPV for women with low economic resources [6–8,30,31]. Women often remain in violent relationships because they are financially unable to leave, which leaves them no option but to stay with a violent partner [7]. The presence of children complicates the situation further: household income per capita is often lower, women’s earnings tend to be reduced due to childcare responsibilities, and the decision to separate becomes even more difficult.

An extension of power resource theory – known as ‘relative resource theory’ – suggests that IPV is more common when there is an imbalance of resources between partners. According to this perspective, “the balance of power will be on the side of that partner who contributes the greater resources to the marriage” or partnership [33]. From a gendered perspective, male power and IPV are intensified when there is a resource imbalance to his disadvantage [47,48]. Following the so-called ‘gendered relative resource theory’ and the perspective of the perpetrator, men may feel their identity is threatened if their female partner has more resources, such as income, education, or prestige (based on Brines’ [49] idea of the gender display approach). This may lead them to react with violence as a way to assert their masculinity, while the female partner may tolerate the violence to conform to traditional gender roles and be seen as ‘a good woman’ and demonstrate female subservience. Atkinson et al. [50] followed the gendered relative resource theory by arguing that the effect of relative resources depends on husbands’ gender ideologies. Using data from the first wave of the National Survey of Families and Households in the U.S., they tested three theories of wife abuse. First, their findings showed no support for the absolute resource theory, which suggests that husbands’ absolute level of resources should be negatively associated with the likelihood of wife abuse. Second, they found limited support for the relative resource theory, which focuses on the relative level of husband’s resources on the likelihood of wife abuse. Third, the strongest support was found for the gendered resource theory: relative resources contribute to IPV only when the male partner holds traditional values and attitudes. Overall, these findings suggest that a comprehensive understanding of IPV necessitates consideration of both attitudes and structural forces.

While power resource approaches highlight structural economic disadvantage as a risk factor for IPV, they do not fully capture the interpersonal dynamics and deliberate strategies of control that often accompany economic abuse. Evan Stark’s theory of coercive control [51,52] addresses this limitation and has become a foundational framework in contemporary IPV research. Stark conceptualizes IPV not merely as episodic acts of physical violence, but as a strategic course of domination aimed at entrapment. Stark defines coercion as “the use of force or threats to compel or dispel a particular response”, whereas control refers to “structural forms of deprivation, exploitation, and command that compel obedience indirectly” [51]. When coercion and control intersect, Stark describes the result as a “condition of unfreedom” that is experienced as entrapment [51]. These mechanisms operate not only through direct violence but through sustained restrictions on a woman’s autonomy, identity, and access to resources. Stark’s guiding hypothesis is that men’s level of coercive control causes women’s overall level of victimization, including the types of physical and sexual violence they suffer. A key element of the interpersonal mechanism of coercive control is economic abuse, which is defined as the control of a partner’s ability to acquire, use, and maintain economic resources. This includes behaviors such as employment sabotage, control over finances, surveillance of spending, and the creation of debt in the partner’s name. From this perspective, economic deprivation is not only a precondition for IPV, but also a product of it. Through coercive control, perpetrators intentionally restrict women’s access to resources and employment, thereby reinforcing dependence and isolation and reducing their capacity to leave the relationship. These practices are not only reflective of broader gender inequalities, but actively reproduce and deepen economic marginalization within the relationship. The coercive control theory thus reveals the bidirectional and dynamic relationship between economic deprivation and IPV: economic deprivation may increase vulnerability to IPV, but IPV – specifically through economic abuse – can also generate or exacerbate economic deprivation.

Stark’s framework encompasses not only physical forms of IPV but also other forms, such as financial and emotional abuse, and thus has a broader perspective than the study at hand. Ideally, this theoretical framework would be tested using dyadic data to account for the relative distribution of power within partnerships. While the *pairfam* dataset does include dyadic information, the sample size for such an analysis is insufficient to reliably test the gendered relative power perspective. However, since this study focuses on physical IPV and its association with economic deprivation, it theoretically relies on the absolute power resource perspective. Given persistent structural inequalities – such as the gender pay gap [53], pension gap [54], and wealth gap [55] – women are, on average, more likely than men to experience economic deprivation, which can also be tested with individual data.

### A power resource approach: An empirical implementation

Following the previously mentioned power resource approaches, empirically, we utilize two indicators of economic dependence: unemployment and concerns about the household’s finances.

First, unemployment serves as an indicator of economic powerlessness. A loss of income limits the ability to finance everyday life without the financial support of a partner. For instance, managing the costs of a house once shared with a partner or moving out and affording a new place independently becomes significantly more difficult without an income. Therefore, we hypothesize that:

*H1: Unemployment increases the risk of IPV.*

Second, income is a factor of economic power. Many studies have examined how poverty affects the risk of violence [e.g., 56,57]. However, these studies often fail to capture the stress associated with poverty [6,7]. The subjective perceptions of economic hardship are a trigger for family stress, aggression, and, in the worst case, violence [e.g., 7]. Therefore, the second hypothesis is:

*H2: Dissatisfaction with the household income increases the likelihood of IPV.*

## Data, variables, and methods

### Data

This study utilizes data from 14 waves of the German Family Panel (release 14.2 [58]). *pairfam* commenced in 2008 with the goal to provide insights into the formation and progression of intimate relationships and families in Germany [59]. The panel collects annual data from a probability-based sample across three birth cohorts: 1971–1973, 1981–1983, and 1991–1993. Additionally, wave 11 introduced a supplementary cohort of respondents born between 2001 and 2003. Data collection was conducted in accordance with the ethical standards for the treatment of human subjects (German Research Foundation, Reference Number 19016KH). *pairfam* was approved by the ethics committee of the Faculty of Management, Economics and Social Sciences of the University of Cologne. Informed consent was obtained verbally from all participants included in the study. Data were accessed from *pairfam* for research purposes between July 1, 2024 and June 30, 2025. Note that authors had no access to any information that could identify individual participants.

### Variables

#### Dependent variable.

When examining intimate partner violence, it is essential to consider the potential for underreporting due to the sensitive nature of the topic. To mitigate this issue, *pairfam* implemented specific measures to enhance privacy during interviews and thus reduce underreporting of IPV. The primary mode of data collection is computer-assisted face-to-face interviewing (note that starting in wave 12, COVID-19-related mode adjustments were made). However, questions concerning violence and other sensitive topics – particularly those susceptible to social desirability bias – were administered via computer-assisted self-interviewing (CASI). Respondents were provided with a computer by the interviewer to independently answer the survey questions, thus eliminating the need for an interviewer to ask them directly. Research indicates that self-administered questionnaires lead to higher disclosure rates of sensitive information and more accurate results [60]. Moreover, respondents had the option to select *‘I do not want to answer that’* as a response. This option not only allowed them to abstain from answering but also helped them avoid disclosing sensitive information about violence, thus preventing the need for a potentially dishonest response.

The specific question coded as our outcome variable is: *‘In the past year up to the point of your separation, were there any arguments between you and your ex-partner during which either of you used physical force?’* Responses are coded as ‘1’ if IPV occurred (either perpetrated by the partner or mutually) and ‘0’ if no IPV was reported (note that we perform a robustness check excluding cases where respondents reported that there were violent encounters between them and their partner, rather than them being solely the survivor of IPV; see ‘Sensitivity analyses’ section). Cases with responses such as ‘I don’t want to answer’ or ‘I don’t know’ are treated as missing values and excluded from the analysis (we conduct an additional robustness check, where we combine cases of ‘I don’t want to answer that’ with those who actually reported experiencing IPV and re-analyze the data; see ‘Sensitivity analyses’ section). Consequently, the dependent variable measures whether respondents experienced IPV in their most recent relationship prior to separation.

#### Independent variables.

The main set of independent variables focuses on respondents’ economic resources. As aforementioned, we include two measures of economic deprivation. First, the satisfaction with the household’s financial situation, ranging from ‘very dissatisfied’ (0) to ‘very satisfied’ (10). Note that the data capture an overall (dis-)satisfaction with the household’s income and do not allow us to disentangle that (dis-)satisfaction further, such as discerning whether it stems for example from frustrations with the partner’s income and a questioning of traditional ‘breadwinner’ norms, or from frustration over one’s own income or inability to earn income or something else. Second, we consider whether the respondent was unemployed for at least one month in the last year.

Several other factors are controlled for in our analysis. First, we account for the number of biological children, categorized as no children, one child, or two or more children. The presence of children may be associated with the likelihood of experiencing IPV through various channels, including increased psychological distress, elevated caregiving demands, the stress associated with balancing work and family responsibilities, and reduced time and energy available for maintaining the couple relationship. Additionally, having children may also serve as a proxy for economic deprivation. For many – though not all – families and more specifically women, children represent a significant financial burden. This can occur through mechanisms such as reducing hours of paid employment, transitioning from full-time to part-time work, or exiting the labor market entirely. These changes can exacerbate economic dependence, which often flows from mother to father in heterosexual households and may increase vulnerability to IPV. Furthermore, we control for several socio-economic variables. Educational level is measured using a categorical dummy variable based on the CASMIN classification, indicating whether the respondent has low, medium, or high education or is currently enrolled. We also include a dummy variable to distinguish respondents in Western and Eastern Germany, accounting for regional differences. Another dummy variable indicates whether the respondent resides in an urban area with a population exceeding 500,000. Respondent’s age is included as a continuous variable, and yearly dummies are incorporated to capture period effects. An overview of all variables is provided in the supporting information (S1 Table).

### Methodological challenges: Determining the exact timing of IPV occurrence

A methodological challenge arises in determining the exact timing of IPV incidents. While the data indicate whether IPV occurred between the previous and current interviews, the precise timing remains unclear. This uncertainty is problematic if key independent variables changed during this period, as it is impossible to ascertain whether these changes preceded or followed the IPV.

To address this issue, we employ two strategies. First, for measuring unemployment – one of our central dependent variables – we utilize event history calendar (EHC) data, which retrospectively tracks individual life course changes on a monthly basis between interview waves, providing detailed information on employment transitions. For all other independent variables, where EHC data are not available, we use lagged values from the previous wave (t-1 or t-2 in the few cases where the yearly interview was missed) to establish a chronological order that logically follows the cause-and-effect relationship (note that measures for satisfaction with household finances are not available in *pairfam’s* first wave; therefore, values from the subsequent interview were used in these cases). Since no information on IPV in the last year is available in wave 1, we pool the data on IPV starting from the second *pairfam* wave and the subsequent waves. Most independent variables are drawn from the interview taking place prior to the information on IPV and dissolution of partnership or from the EHC. Thus, information on IPV is retrieved from *pairfam* waves 2–14, while most independent variables are retrieved from waves 1–13 or the EHC (for a similar approach using the retrospective data collected by *pairfam* on abortions, see [61]). Note that *pairfam* also includes a question on IPV in the current relationship. However, since this question is asked only biennially from wave 3 onwards, we refrain from using these data in our main analysis due to the methodological challenges posed by the time lag. Bridging two or more waves, rather than just one, would likely yield imprecise estimates. Nonetheless, we conduct an additional robustness check using these data.

### Sample

After pooling all available waves of data from the base *pairfam* sample and its refreshment, our sample includes 17,423 respondents (94,081 respondent-years). The question on intimate partner violence is specifically worded to include only respondents who were in a relationship and separated between the last and current wave (see above for the exact wording of the item). Consequently, we can only analyze the responses of those who reported a dissolution of their partnership since the last survey interview, as no valid observations are available for others. After deleting these respondents and those with missing information on the IPV variable, we lose 77,165 respondent-years. We also drop all first interviews, resulting in a loss of 12,402 respondent-years. This step is necessary because, as previously mentioned, in the first year of the survey, respondents were not asked whether they had experienced IPV in the past year. Instead, they were administered a more general question that asked whether they had ever experienced IPV at any point in their lives. Since we are only interested in women, we retain only female respondents in our sample, which results in a loss of 2,045 respondent-years. We also exclude 12 cases of women who reported being the sole perpetrator in an IPV incident. Finally, we lose a further 14 cases due to missing values for the independent variables. In total, we end up with a sample of 1,667 respondents and 2,443 respondent years with 272 IPV incidents.

### Methods

First, we conduct a descriptive analysis of intimate partner violence. Next, we examine the relationship between IPV and our independent variables using linear probability models (LPM [62]). To account for the panel structure of the *pairfam* data and the pooling of fourteen survey waves, we estimate robust standard errors clustered at the individual level. Instead of using the survey weights provided by *pairfam*, we incorporate relevant socio-economic variables as controls in the multivariate analysis (see description of control variables above). Finally, we perform several sensitivity checks to assess the robustness of our results. While our extensive robustness analyses strengthen the credibility of our findings – and may bring us closer to causal inference if causality is understood as a continuum rather than a binary state – they are certainly not sufficient to warrant what is conventionally regarded as causal conclusions. The observed relationships should therefore be interpreted as associations rather than causal effects.

## Results

### Descriptive findings

**Fig 1:**
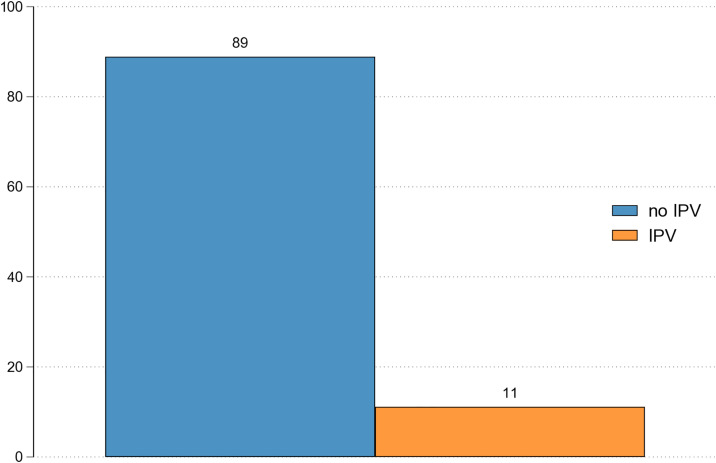
IPV before the dissolution of the partnership. Note: Based on *pairfam* 14.2, own calculations, not weighted.

In our sample, we find that 272 female respondents reported experiencing IPV, either solely from their partner or through violent altercations with their partner prior to the end of the relationship. In contrast, 2,171 respondents did not experience IPV before splitting up. Thus, 11 percent of all women in our sample who reported a dissolution of partnership also reported IPV, either by them or by both, themselves and their partner (see Fig 1). Note that Fig 1 is not weighted, as *pairfam* does not provide a specific weight for our analytical sample (i.e., the combined base and refreshment samples). While a combined weight is available for the base, refreshment, and demodiff samples, we had to exclude demodiff respondents due to missing data. However, when applying these available weights to our sample (results not shown), the estimate increases to approximately 13% of women reporting IPV around the time of union dissolution. This suggests that the unweighted figure presented in Fig 1 may in fact be conservative.

### Multivariate analysis: Main model

**Fig 2:**
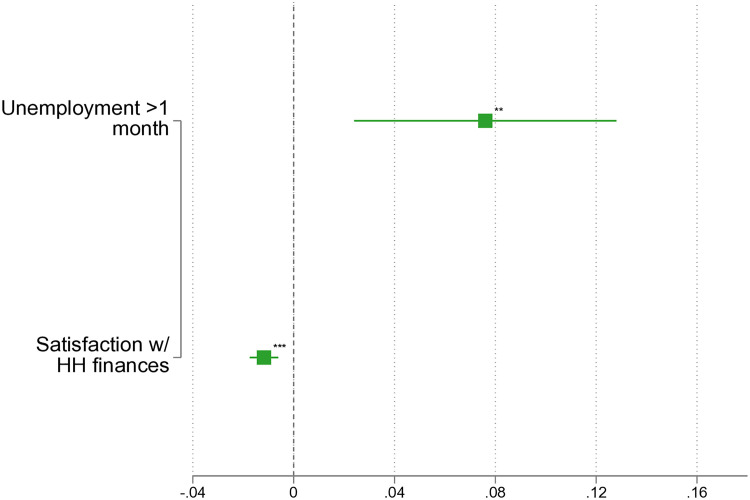
LPM: IPV before the dissolution of the partnership. Note: Based on *pairfam* 14.2, own calculations, not weighted. ^*^ *p* < 0.05, ^**^ *p* < 0.01, ^***^ *p* < 0.001. Full results in S2 Table.

Fig 2, with full results available in S2 Table, illustrates the association between economic factors and IPV. Notably, the data indicate that unemployment between the last and current interviews is associated with an increased risk of IPV for women by 7.6 percentage points, supporting Hypothesis 1 (*H1*). Furthermore, Fig 2 reveals that a one-point increase on a elven-point scale (0-10) measuring satisfaction with household finances is associated with a 1 percentage point reduction in the risk of IPV. Therefore, complete satisfaction with household finances – indicated by selecting the highest score of 10 – is associated with a reduced risk of IPV of about 10 percentage points, aligning with Hypothesis 2 (*H2*).

Additionally, turning to the control variables, our findings indicate that having children is significantly associated with an increased risk of experiencing IPV (see S2 Table). Compared to individuals without children, having one child is associated with a 10 percentage point increase in risk, while having two or more children is associated with a 13 percentage point increase in risk. This supports our main finding that economic deprivation is associated with a higher risk of IPV. Children can contribute to this dynamic by imposing additional financial strain on families – particularly in cases where caregiving responsibilities reduce women’s labor market participation and increase their economic dependence. This increased economic pressure may, in turn, heighten vulnerability to IPV. However, this economic channel is only one of several pathways through which the presence of children may be associated with IPV (see discussion in the ‘variable’ section). Additionally, the full model includes several other control variables, with results detailed in S2 Table. The table shows that a low level of education is associated with a higher risk of experiencing IPV compared to a medium level of education (see also [9]). This finding further highlights the relevance of economic disadvantage as a contributing factor to IPV. Results also indicate that the prevalence of IPV is associated with a decrease with increasing age. However, we find no significant association between urban residency and IPV, nor between living in Eastern Germany and IPV.

In addition to the analysis detailed above and given our decision not to apply survey weights in the regression analysis, we conduct a supplementary analysis in which we include cohort dummies to account for the cohort-based design of *pairfam* (results not shown). The coefficients for unemployment and satisfaction with household finances remain statistically significant and virtually unchanged in size. We therefore opt to retain the more parsimonious model specification without cohort dummies.

### Sensitivity analysis: Alternate outcome

**Fig 3:**
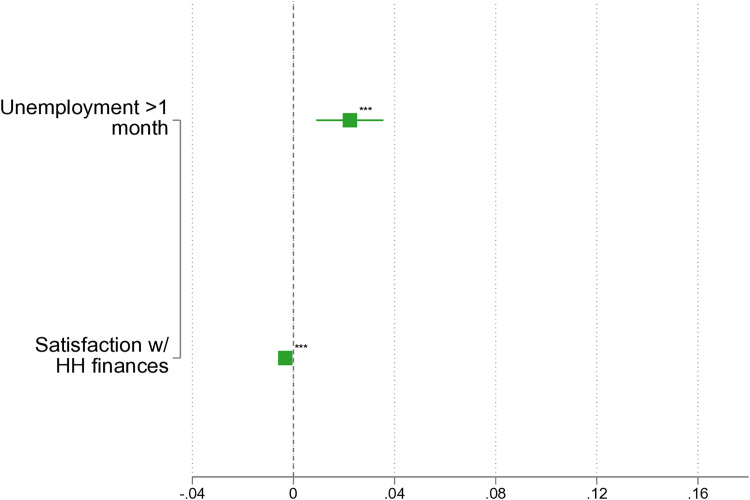
LPM: IPV in current partnerships. Note: Based on *pairfam* 14.2, own calculations, not weighted. ^*^ *p* < 0.05, ^**^ *p* < 0.01, ^***^ *p* < 0.001. Full results in S3 Table.

In our previous analysis, the specific wording and filtering of the outcome measure resulted in the use of only a small fraction of the *pairfam* sample. To enhance the robustness of our findings, we re-analyze the data using an alternative item measuring IPV in the current relationship. Fig 3 shows that when examining current relationships, the results remain comparable in direction and significance to those from past relationships. Unemployment is associated with a higher risk of IPV, while satisfaction with the household’s financial situation acts as a protective factor.

However, it is important to note that the IPV question used in this analysis – unlike the one employed in the main analysis – is only administered every two years, starting from wave 3. This change complicates the temporal alignment of independent variables. Nevertheless, we chose to maintain our original approach of using independent variables from the previous interview, even though this is less precise when the reference period for IPV spans two years rather than one (on average). As a result, this robustness check is inherently less accurate and more susceptible to error compared to our main estimation strategy, which only required bridging the interval between the current and last interview. Nevertheless, the robustness analysis corroborates that unemployment and financial worries remain associated with IPV experiences. These findings underscore the critical role that economic deprivation plays in IPV experiences.

### Sensitivity analysis: Addressing the social desirability bias

**Fig 4:**
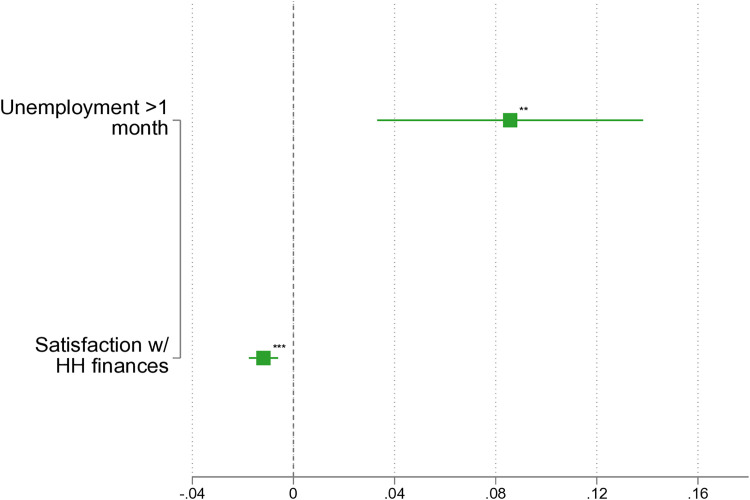
LPM: IPV before the dissolution of the partnership (including ‘I don’t know’). Note: Based on *pairfam* 14.2, own calculations, not weighted. ^*^ *p* < 0.05, ^**^ *p* < 0.01, ^***^ *p* < 0.001. Full results in S4 Table.

Another critical issue in researching intimate partner violence is the influence of social desirability. Respondents may be reluctant to disclose personal and intimate information, which can lead to underreporting of IPV incidents in surveys. To partially address this concern, we include respondents who answered ‘I do not want to answer’ to the IPV question alongside those who reported experiencing IPV. Both groups are now coded as ‘1’ in the binary dependent variable. The results of this adjustment are presented in Fig 4. Overall, we find that the magnitude and strength of the results remain broadly similar to those shown in Fig 2. Based on these findings, we conclude that our analysis generally provides reliable results, despite the challenges posed by social desirability bias.

### Further sensitivity analysis

To further test the robustness of our results, we perform five additional sensitivity checks.

First, respondents who indicated that both partners had engaged in physical violence were excluded from the analysis. As with the current sample, we might also include actions by women that may be in response to prior aggression or aimed at self-protection. Such actions may differ in nature from the physical violence typically observed in heterosexual relationships, where men are statistically more likely to be the primary aggressors and male-perpetrated IPV is associated with higher levels of injury and lethality. This analysis thus focuses solely on female respondents who reported experiencing IPV exclusively from their partner. The results from this adjustment are consistent with those presented in the main body of the paper and can be found in S5 Table.

Second, to ensure that our findings hold under a modeling technique specifically designed for binary outcome variables, we re-run our model using logistic regression. The average marginal effects are reported in S6 Table. The results are broadly similar to those obtained using the LPM method in S2 Table. Additionally, we assess the extent of out-of-range predictions – that is, probabilities below 0 or above 1 – when modeling IPV experiences. Based on the main model presented in S2 Table, only 171 out of 2,443 observations (7% of the sample) yielded out-of-range predicted values. This relatively low proportion further supports the suitability of the linear probability model for our analysis.

Third, to assess the robustness of our results while accounting for time-constant unobserved individual heterogeneity, we re-estimate our model using a linear probability model with a two-way fixed effects estimator. The results, presented in S7 Table, show that unemployment is associated with a ten percentage point increase in the risk of experiencing IPV, largely in line with our main model. The association between satisfaction with household finances and IPV remains stable in size but becomes insignificant. However, given the relatively low incidence of IPV in our dataset, fixed effects estimation poses limitations. Specifically, since fixed effects rely on within-individual variation (i.e., deviations from the individual mean, or ‘de-meaning’), the estimator requires substantial intra-individual change over time – something that is limited in our sample. For this reason, we do not rely on fixed effects as our primary estimation strategy and consider the results in S7 Table largely in line with our main findings.

Fourth, IPV is also known to depend on childhood experiences [e.g., 63,64]. Therefore, we include an additional independent variable in our models that indicates how happy the respondent’s childhood was on a scale of 0–10. Since this variable was only measured once for each respondent, we assume it to be constant over time and insert it for all missing waves. The results remain stable against the inclusion of this variable, but we do find that the happier the childhood, the lower the association with IPV experiences in adulthood (see S8 Table).

Fifth, we re-estimate our main model using a more restrictive definition of unemployment. Specifically, we recode the unemployment variable so that it takes the value of one only if the respondent had been unemployed for three or more months since the last interview; excluding those who experienced only one or two months of unemployment during that period. In our original model, the dummy variable was coded as one if a person had been unemployed for one month or more. We increase the threshold in this robustness check to better capture more severe cases of unemployment, which are likely to be associated with higher levels of financial strain – particularly for women. The results, presented in S9 Table, show that being unemployed for three or more months is associated with an increase in the probability of experiencing IPV by approximately 8.6 percentage points. This is higher than our original estimate of 7.6 percentage points, suggesting that our initial results may in fact be conservative.

Sixth, since our key independent variables (unemployment and satisfaction with household finances) correlate as they both act as proxies for economic deprivation, we conduct two additional regressions including these variables separately; the results can be found in S10 Table. As anticipated, the associations for the two key independent variables with IPV increase slightly, particularly for the variable on unemployment. However, since deviations are not large and the variance inflation factor in the main analysis remains lower than 5 for all the independent variables, we opt to retain the model that includes both key variables simultaneously in our main analysis. Our main results may be considered conservative, as they reflect smaller estimates than would be observed if the variables were included separately (see S10 Table).

## Conclusion and discussion: Economic deprivation matters!

Most research on IPV in Germany has focused on the relationship between violence and health from public health or criminological perspectives, rather than within the context of sociological inequality research. To date, the interplay between violence, work, and socio-economic inequality in the German context has been underexplored, particularly in studies using probability-based survey data. This study fills this gap. Based on the German Family Panel *pairfam*, a large-scale probability-based panel, this article investigated to what extent economic deprivation increases the likelihood of experiencing IPV. The results reveal that economic deprivation is significantly associated with an increased likelihood of IPV for women in Germany. More specifically, unemployment and dissatisfaction with household income are are associated with an increase in the risk of IPV. These findings underscore the relevance of power constellations, particularly in the form of economic deprivation of the female survivor, in explaining IPV in Germany.

Despite its contributions, the study has some limitations. First, we cannot claim causality from our results. Future research would benefit from longer-running panel data, which would offer greater statistical power to more robustly examine the relationship in question. However, a series of robustness checks has confirmed our main findings. Second, underreporting remains a concern. As benchmark statistics on the true extent of IPV are scarce, we are not able to verify the number of incidents of IPV found in this study. Third, the analysis is limited to a pooled cross-sectional sample of female survivors who have left violent relationships, so the findings cannot be generalized to all German couples. However, we believe that our results are likely conservative, as women who leave violent relationships may experience less economic dependence (after all, they *can* leave the relationship) on their partner compared to those who remain in abusive relationships. Therefore, we may be underestimating the relationship between IPV and economic deprivation in Germany.

Notwithstanding these limitations, our study is one of the few to highlight the significance of economic deprivation for the risk of IPV in Germany, using large-scale probability-based panel data, and thus provides insights into the previously under-researched topic of economic deprivation and IPV in Germany. The findings are consistent with research from other countries, such as Reichel [8] in the EU, Lucero et al. [7] in the U.S., and Ahmadabadi et al. [31] in Australia, and underscore the theoretical insights of power resource theory in explaining how economic deprivation is associated with increased vulnerability to IPV. Our study aligns with these works as one of the few to examine the complex relationship between economic deprivation and IPV using large-scale panel data. Both this nexus and the broader causal mechanisms underlying IPV remain insufficiently explored and should be focused on in future research efforts. While Stark’s [51] bidirectional and dynamic perspective on the relationship between economic control and IPV is theoretically relevant, it is not empirically addressed in our study. A closer empirical investigation of this framework could yield further valuable insights.

Beyond its scientific relevance, the study also has important practical implications. A deeper understanding of the relationship between violence and economic deprivation is also indispensable for other countries, in which IPV is a widespread but under-researched issue, and for policy-making processes. Our findings support the objectives of the Istanbul Convention, particularly the requirement to collect data on gender-based violence to monitor the implementation of state obligations and assess the effectiveness of related measures. Moreover, the results underscore important policy implications, including the provision of financial assistance to female IPV survivors, financial literacy programs, and workplace protections such as those promoted by ILO Convention 190. These findings emphasize the urgent need for targeted financial support for female IPV survivors to reduce their risk of violence – even in affluent societies.

## Supporting information

S1 Table Sample characteristics. Descriptive statistics.
(DOCX)

S2 Table IPV before the dissolution of the partnership. Linear probability model.
(DOCX)

S3 Table IPV in current partnerships. Linear probability model.
(DOCX)

S4 Table IPV before the dissolution of the partnership: Including ‘I don’t know’. Linear probability model.
(DOCX)

S5 Table IPV before the dissolution of the partnership: Excluding mutual perpetrators. Linear probability model.
(DOCX)

S6 Table IPV before the dissolution of the partnership. Logistic regression, reported are average marginal effects.
(DOCX)

S7 Table IPV before the dissolution of the partnership. Linear probability model with two-way fixed effects.
(DOCX)

S8 Table IPV before the dissolution of the partnership: Including childhood experiences as additional IV. Linear probability model.
(DOCX)

S9 Table IPV before the dissolution of the partnership: Unemployment ≥ 3 months. Linear probability model.
(DOCX)

S10 Table IPV before the dissolution of the partnership: Separate regressions for key dependent variables. Linear probability model.
(DOCX)

S1 Materials Stata syntax. (ZIP)
