# Supplementary material for: Economic deprivation and intimate partner violence in Germany
Source: PLoS One. 2025 Aug 18;20(8):e0329930. doi: 10.1371/journal.pone.0329930 (PMC12360516; doi:10.1371/journal.pone.0329930)
Supplement: S1 Table — Descriptive statistics. (DOCX) [file pone.0329930.s001.docx]

# S1 Table. Sample characteristics. Descriptive statistics.

|  | Mean | SD |
| --- | --- | --- |
| IPV | 0.11 | (0.31) |
| Unemployment | 0.11 | (0.31) |
| Satisfaction w/ HH finances | 6.49 | (2.60) |
| No children | 0.75 | (0.43) |
| One child | 0.12 | (0.32) |
| Two or more children | 0.13 | (0.33) |
| **Controls** |  |  |
| Low education | 0.11 | (0.31) |
| Intermediate education | 0.32 | (0.47) |
| High education | 0.12 | (0.32) |
| Currently enrolled | 0.46 | (0.50) |
| Age | 25.1 | (8.30) |
| Urban >500,000 inhabitants (=1) | 0.15 | (0.36) |
| Living in East Germany (=1) | 0.22 | (0.42) |
| Respondents | 1,667 | |
| Respondent-years | 2,443 | |

Note: Based on *pairfam* 14.2, standard deviation in parentheses, own calculations, not weighted.
