# Supplementary material for: Economic deprivation and intimate partner violence in Germany
Source: PLoS One. 2025 Aug 18;20(8):e0329930. doi: 10.1371/journal.pone.0329930 (PMC12360516; doi:10.1371/journal.pone.0329930)
Supplement: S7 Table — Linear probability model with two-way fixed effects. (DOCX) [file pone.0329930.s007.docx]

**S7 Table. IPV before the dissolution of the partnership.** Linear probability model with two-way fixed effects.

|  | IPV | | |
| --- | --- | --- | --- |
| Unemployment | 0.10^*^ | (0.04) | |
| Satisfaction w/ HH finances | -0.01 | (0.01) | |
| No children | *Ref.* | | |
| One child | -0.02 | (0.08) | |
| Two or more children | -0.03 | (0.17) | |
| **Controls** |  |  | |
| Low education | 0.19^*^ | (0.09) | |
| Intermediate education | *Ref.* | | |
| High education | 0.03 | (0.05) | |
| Currently enrolled | 0.03 | (0.03) | |
| Age | -0.00 | (0.04) | |
| Urban >500,000 inhabitants (=1) | 0.11 | (0.06) | |
| Living in East Germany (=1) | 0.07 | (0.08) | |
| Yearly dummies | ✓ | | |
| Constant | 0.16 | | (0.71) |
| Observations-years | 2,443 | | |
| Observations (persons) | 1,667 | | |
| Within R^2^ | 0.05 | | |

Note: Based on *pairfam* 14.2, individual cluster robust standard errors in parentheses, own calculations, not weighted. ^*^ *p* < 0.05, ^**^ *p* < 0.01, ^***^ *p* < 0.001.
