# Supplementary material for: Economic deprivation and intimate partner violence in Germany
Source: PLoS One. 2025 Aug 18;20(8):e0329930. doi: 10.1371/journal.pone.0329930 (PMC12360516; doi:10.1371/journal.pone.0329930)
Supplement: S10 Table — Linear probability model. (DOCX) [file pone.0329930.s010.docx]

# S10 Table. IPV before the dissolution of the partnership: Separate regressions for key IVs. Linear probability model.

|  | IPV (M1: only unemployment) | | | | IPV (M1: only sat. w/ household finances) | |
| --- | --- | --- | --- | --- | --- | --- |
| Unemployment | 0.09^***^ | | (0.03) | | *X* | |
| Satisfaction w/ HH finances | *X* | | | | -0.01^***^ | (0.00) |
| No children | *Ref.* | | | | *Ref.* | |
| One child | 0.11^***^ | (0.03) | | | 0.10^***^ | (0.03) |
| Two or more children | 0.13^***^ | (0.03) | | | 0.14^***^ | (0.03) |
| **Controls** |  |  | | |  |  |
| Low education | 0.12^***^ | | (0.03) | | 0.12^***^ | (0.03) |
| Intermediate education | *Ref.* | | | | *Ref.* | |
| High education | -0.01 | | (0.02) | | -0.00 | (0.02) |
| Currently enrolled | -0.02 | | (0.02) | | -0.02 | (0.02) |
| Age | -0.01^***^ | | (0.00) | | -0.01^***^ | (0.00) |
| Urban >500,000 inhabitants (=1) | -0.00 | | (0.02) | | 0.00 | (0.02) |
| Living in East Germany (=1) | 0.00 | | (0.02) | | 0.01 | (0.02) |
| Yearly dummies | ✓ | | | | ✓ | |
|  | 0.28^***^ | | | (0.04) | 0.38^***^ | (0.05) |
| Observations | 2,443 | | | | 2, 443 | |
| R^2^ | 0.07 | | | | 0.07 | |

Note: Based on *pairfam* 14.2, individual cluster robust standard errors in parentheses, own calculations, not weighted. ^*^ *p* < 0.05, ^**^ *p* < 0.01, ^***^ *p* < 0.001.
